# Supplementary material for: Tau P301S Transgenic Mice Develop Gait and Eye Movement Impairments That Mimic Progressive Supranuclear Palsy
Source: bioRxiv. 2024 Sep 23:2024.09.20.614197. Preprint. [Version 1] doi: 10.1101/2024.09.20.614197 (PMC11463522; doi:10.1101/2024.09.20.614197)
Supplement: Supplementary file 1 [file media-1.pdf]

SUPPLEMENTAL FIGURES AND TABLES

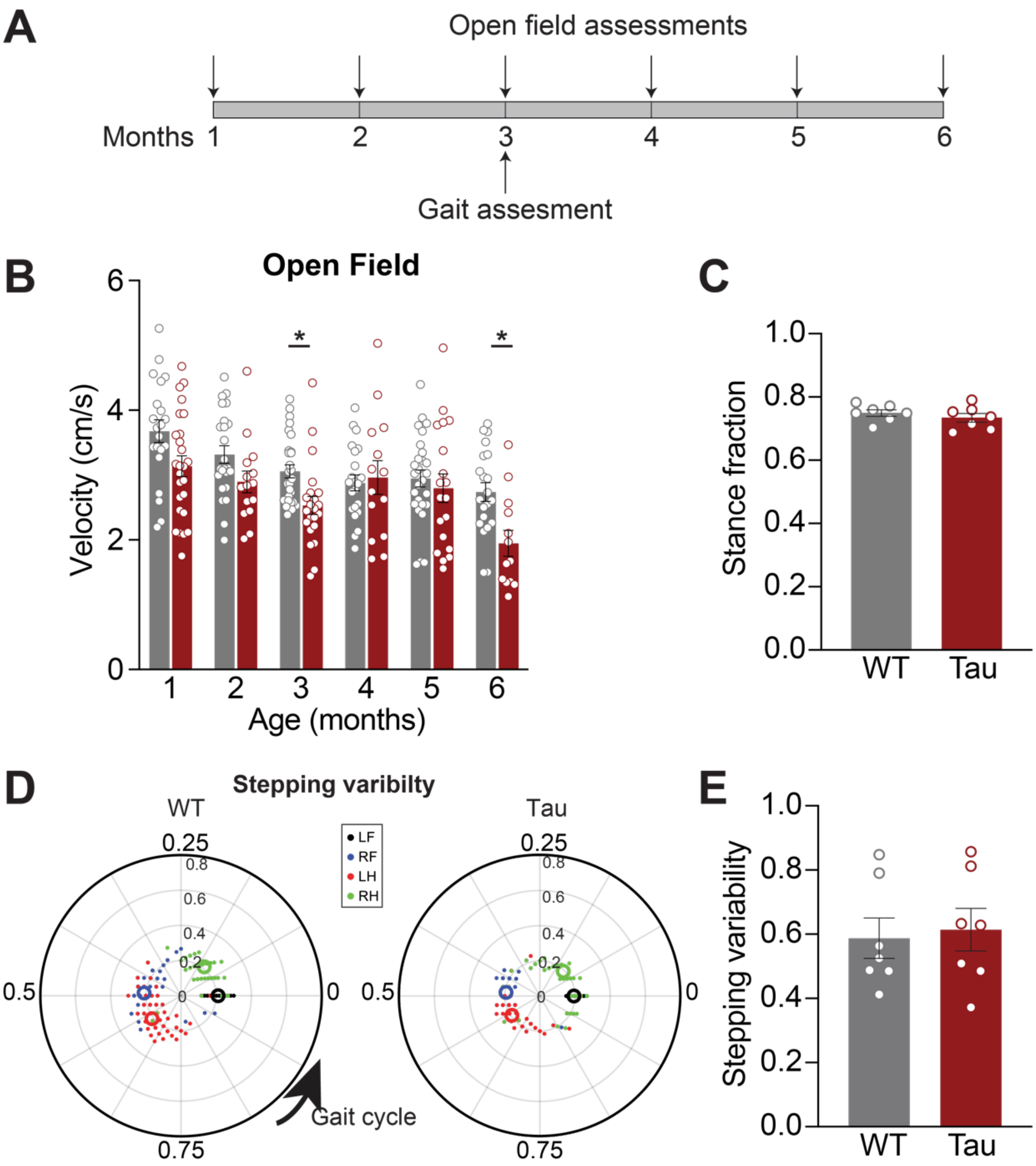

Figure 1 Supplemental (associated with Figure 1). At 3 months of age, Tau mice do not show impairments in locomotor coordination.

A. Experimental timeline for open field assessment and gait analysis on the linear track. **B.** Average open field locomotor velocity of Tau and WT mice. Tau mice moved more slowly at 3 (N = 28 WT, 22 Tau, p = 0.0246) and 6 months (N = 21 WT, 13 hP301S p = 0.0236). **C.** Average stance fraction in 3-month-old WT and Tau mice (N = 7 WT, 7 Tau, p = 0.5350). **D.** Polar plots indicating phase of the gait cycle where each limb enters stance, aligned to the stance onset of the left forelimb. Individual dots represent a single stride, different colors correspond to different limbs (LF- left forelimb, RF- right forelimb, LH- left hindlimb, RH- right hindlimb). The distance between the center and the dots represents the duration (s) of that stride. Larger open circles represent the average stride for each limb. **E.** Quantification of stepping variability (standard deviation) across limbs in 3-month-old WT and Tau mice (N = 7 WT, 7 Tau, p = 0.7104). N refers to mice. Data is shown as mean +/-SEM. Overlaid open circles in B, C, E represent individual animals.

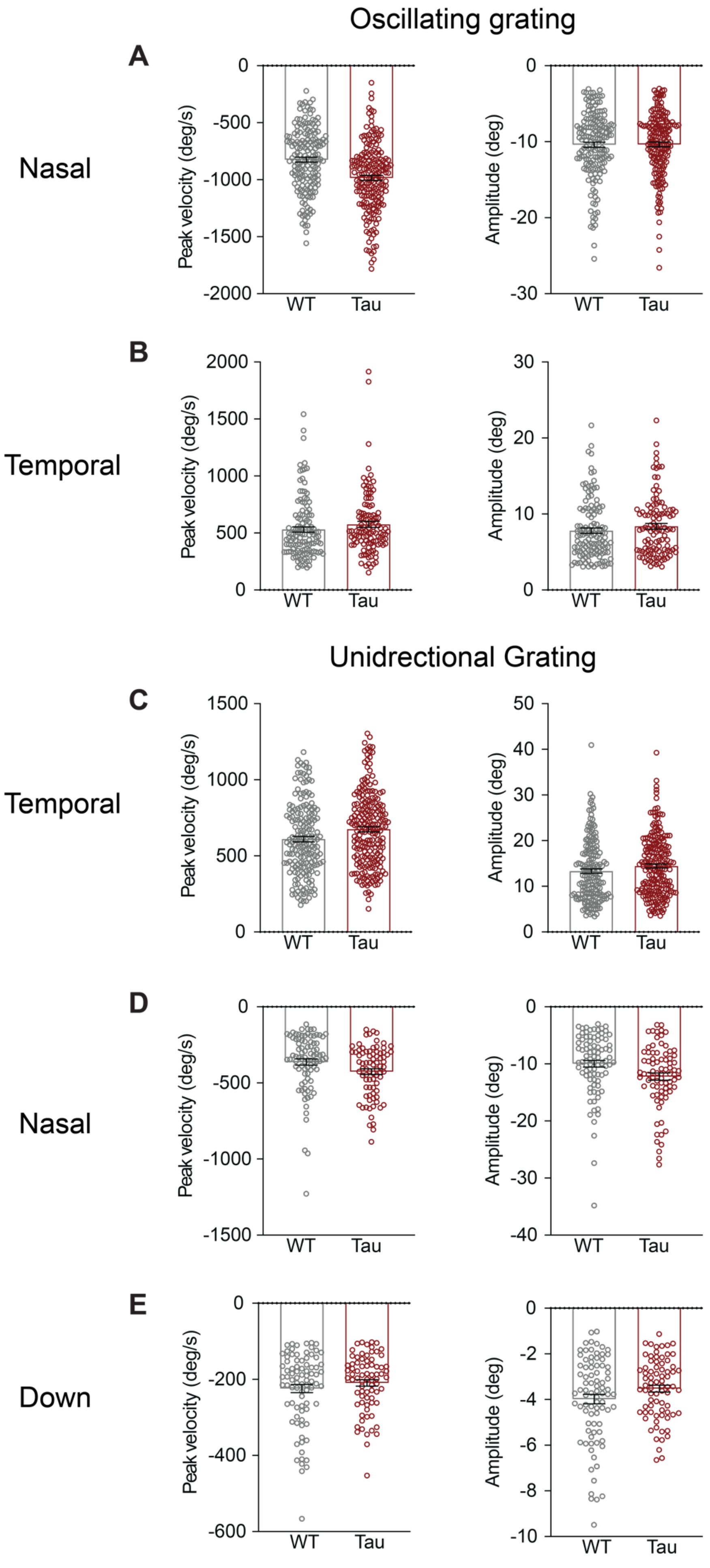

Oscillating grating
A
Nasal
Peak velocity (deg/s)
0
-500
-1000
-1500
-2000
WT
Tau
Amplitude (deg)
0
-10
-20
-30
B
Temporal
2000
1500
1000
500
30
20
10
Unidrectional Grating
C
1500
50
40
D
-40
E
Down
-200
-400
-600
-2
-4
-6
-8

Figure 2 Supplemental (Associated with figure 3 and 4).
A. Peak velocity (left) and amplitude (right) of nasal spontaneous saccade-like eye movements made during stimulation with a horizontally oscillating grating. WT: N = 9, n = 170, Tau: N = 7, n = 191. **B**. Peak velocity (left) and amplitude (right) of temporal spontaneous saccade-like eye movements made during stimulation with the same oscillating grating WT: N = 9, n = 125, Tau: N = 7, n = 111. **C.** Peak velocity (left) and amplitude (right) of temporal resetting saccades made during stimulation with the horizontal unidirectional grating. WT: N = 8, n = 184, Tau: N = 6, n = 214. **D.** Peak velocity (left) and amplitude (right) of nasal resetting saccades made during stimulation with the horizontal unidirectional grating. WT: N = 8, n= 88, Tau: N = 6, n = 80. **E.** Peak velocity (left) and amplitude (right) of downward resetting saccades made in response to the vertical unidirectional grating. WT: N = 8, n= 84, Tau: N = 6, n = 76. Each open circle represents a single resetting saccade. Data is shown as mean +/- SEM. N = mice, n = saccades.

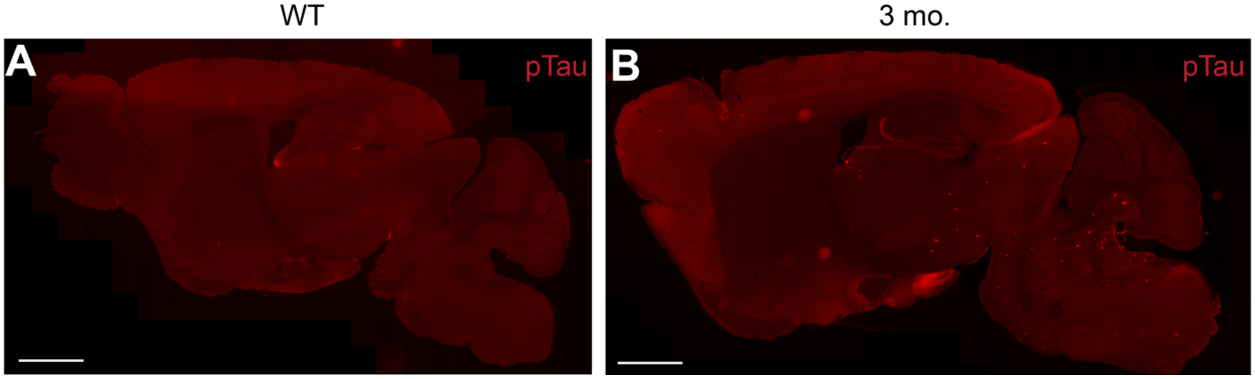

Supplementary Figure 3 (associated with figure 5). Tau immunoreactivity in WT and 3 mo. old Tau mice.

A. Representative sagittal section from a 5-month-old WT mouse stained for phosphorylated Tau. **B.** Representative sagittal section from a 3-month-old Tau transgenic mouse stained for phosphorylated Tau. Scale bar = 1mm.

**Table S1: GAIT PARAMETERS ANALYSIS AND STATISTICS FOR 5 MO. OLD MICE**<br>Mann Whitney U test was used for all comparisons.<br>Table shows statistics for both comparisons using either individual stride or stride averages across mice					
	WT (N = mice, n = strides)	hP301S mice (N = mice, n = strides)	Medians<br>First value- n median<br>Second value () - N median	Comparisons of individual strides	Comparison of individual animals
Stride length	N = 16, n = 364	N = 25, n = 707	WT: 3.678 (3.537)<br>hP301S: 3.171 (3.344)	P < 0.0001	P = 0.1322
Cadence (steps/ sec)			WT: 2.609 (2.640)<br>hP301S: 2.857 (2.849)	P < 0.0001	P= 0.0434
Speed (cm/s)			WT: 8.102 (8.094)<br>hP301S: 8.241 (8.748)	P = 0.5854	P = 0.7613
Stace fraction			WT: 0.6667 (0.6734)<br>hP301S: 0.7273 (0.7401)	P < 0.0001	P = 0.0013 (Fig. 1E)
Swing fraction			WT: 0.3810 (0.3670)<br>hP301S: 0.3182 (0.3117)	P < 0.0001	P = 0.0018

Table S2: Correlations of Tau pathology density to locomotor and oculomotor impairments in 5-6 mo. old Tau mice.

Spearman correlation was used for all brain regions
Bonferroni Corrected p-values:
Rotarod and Stepping variability – $p < 0.006$
Vertical quick phases – $p < 0.01$

Brain Region	Rotarod (latency to fall)	Stepping variability (STD)	Vertical quick phase frequency
Motor Cortex	r = -0.7939<br>p = 0.0088	r = 0.5779<br>p = 0.0525	N/A
Subthalamic Nucleus	r = -0.0424<br>p = 0.9184	r = 0.4476<br>p = 0.1474	N/A
Zona Incerta	r = -0.6848<br>p = 0.0347	r = 0.6643<br>p = 0.0219	r = 0.5798<br>p = 0.2444
Substantia Nigra pars reticulata	r = -0.3212<br>p = 0.3679	r = 0.5105<br>p = 0.0936	r = 0.3189<br>p = 0.5444
Pedunculopontine Nucleus	r = -0.7333<br>p = 0.0202	r = 0.6993<br>p = 0.0142	N/A
Cuneiform Nucleus	r = -0.5273<br>p = 0.1231	r = 0.3566<br>p = 0.2560	N/A
Superior Colliculus (medial)	N/A	N/A	r = 0.1160<br>p = 0.8444
Medial Vestibular Nucleus	r = -0.9152<br>p = 0.0005	r = 0.4406<br>p = 0.1542	r = 0.5444<br>p = 0.3189
Deep Cerebellar Nuclei	r = -0.6606<br>p = 0.0438	r = 0.6364<br>p = 0.0299	r = 0.8697<br>p = 0.0333

**Table S3. EXPERIMENTAL ANALYSIS AND STATISTICS**<br>MWU- Mann- Whitney U test; RM-ANOVA- repeated measures analysis of variance						
**Key Experiments**	**Figure**	**Comparison**	**Statistical test**	**N (animals)**	**n (strides or events)**	***p* value**
Longitudinal Rotarod analysis	Fig. 1B	Between group	Mixed-effects analysis (Sidak's multiple comparison's test)	WT:<br>1 mo. – 30<br>2 mo. – 30<br>3 mo. – 28<br>4 mo. – 20<br>5 mo. – 21<br>6 mo. – 22<br><br>hP301S:<br>1 mo. – 38<br>2 mo. – 27<br>3 mo. – 22<br>4 mo. – 14<br>5 mo. – 13<br>6 mo. – 15	N/A	1 mo. – p > 0.9<br>2 mo. – p > 0.5<br>3 mo. – p > 0.1<br>4 mo. – p > 0.9<br>5 mo. – p > 0.9<br>6 mo. – p < 0.0001
5 mo. Stance Fraction	Fig. 1F	Between group	MWU	WT = 16<br>hP301S = 25	N/A	p = 0.0013
5 mo. Stepping Variability	Fig. 1H	Between group	MWU	WT = 16<br>hP301S = 25	N/A	p = 0.0002
Longitudinal open field analysis	Fig. S1B	Between group	Mixed-effects analysis (Sidak's multiple comparison)	WT:<br>1 mo. – 21<br>2 mo. – 24<br>3 mo. – 28<br>4 mo. – 22<br>5 mo. – 27<br>6 mo. – 21<br><br>hP301S:<br>1 mo. – 26<br>2 mo. – 15<br>3 mo. – 22<br>4 mo. – 14<br>5 mo. – 19<br>6 mo. – 13	N/A	1 mo. – p > 0.1<br>2 mo. – p > 0.3<br>3 mo. – p = 0.0247<br>4 mo. – p > 0.9<br>5 mo. – p > 0.9<br>6 mo. – p = 0.0236
3 mo. Stance Fraction	Fig. S1C	Between group	MWU	WT = 7<br>hP301S = 7	N/A	p = 0.5350
3 mo. Stepping Variability	Fig. S1E	Between group	MWU	WT = 7<br>hP301S = 7	N/A	p = 0.7104
OKR gain	Fig. 2D	Between group	Mixed-effects analysis (w/ Sidak's multiple comparisons)	WT = 9<br>hP301S = 7	N/A	0.2: p = 0.4183<br>0.4: p = 0.6412<br>0.6: p = 0.9955<br>0.8: p = 0.9970<br>1.0: p = 0.9750
Oscillating drum: Nasal main sequence	Fig. 3D	N/A	N/A	WT = 9<br>hP301S = 7	WT = 170<br>hP301S = 191	N/A

Oscillating drum: Temporal Main sequence	Fig. 3E	N/A	N/A	WT = 9<br>hP301S = 7	WT = 111<br>hP301S = 125	N/A
OKR Gain	Fig. 4D	Between group	Two-way RM ANOVA (with Bonferroni correction)	WT = 8<br>hP301S = 6	N/A	T: p = 0.5185<br>N: p > 0.9999<br>V: p = 0.4430<br>D: p = 0.8834
Linear drum: Temporal main sequence	Fig. 4E	N/A	N/A	WT = 8<br>hP301S = 6	WT = 184<br>hP301S = 214	N/A
Linear drum: Nasal main sequence	Fig. 4F	N/A	N/A	WT = 8<br>hP301S = 6	WT = 88<br>hP301S = 80	N/A
Linear drum: Ventral main sequence	Fig. 4G	N/A	N/A	WT = 8<br>hP301S = 6	WT = 84<br>hP301S = 76	N/A
Nasal Quick phase	Fig. 4H	Between group	MWU	WT = 8<br>hP301S = 6	N/A	p = 0.8248
Temporal Quick phase	Fig. 4I	Between group	MWU	WT = 8<br>hP301S = 6	N/A	p = 0.1678
Ventral Quick phase	Fig. 4J	Between group	MWU	WT = 8<br>hP301S = 6	N/A	p = 0.0373
Dorsal Quick phase	Fig. 4K	Between group	MWU	WT = 8<br>hP301S = 6	N/A	p = 0.0077
OKR Gain	Fig. 4D	Between group	Two-way RM ANOVA (with Bonferroni correction)	WT = 8<br>hP301S = 6	N/A	T: p = 0.5185<br>N: p > 0.9999<br>V: p = 0.4430<br>D: p = 0.8834
Oscillating grating: Nasal	Fig. S2A	Between group	MWU	WT = 8<br>hP301S = 6	WT = 170<br>hP301S = 191	Velocity: p = 0.3490<br>Amplitude: p = 0.7546
Oscillating grating: Temporal	Fig. S2B	Between group	MWU	WT = 8<br>hP301S = 6	WT = 111<br>hP301S = 125	Velocity: p = 0.9497<br>Amplitude: p = 0.8518
Unidirectional grating: Temporal	Fig. S2C	Between group	MWU	WT = 8<br>hP301S = 6	WT = 184<br>hP301S = 214	Velocity: p = 0.9497<br>Amplitude: p = 0.8518
Unidirectional grating: Nasal	Fig. S2D	Between group	MWU	WT = 8<br>hP301S = 6	WT = 88<br>hP301S = 80	Velocity: p = 0.4908<br>Amplitude: p = 0.4448
Unidirectional grating: Down	Fig. S2E	Between group	MWU	WT = 8<br>hP301S = 6	WT = 84<br>hP301S = 76	Velocity: p = 0.7546<br>Amplitude: p = 0.7546
